# Integration site–dependent HIV-1 promoter activity shapes host chromatin conformation

**DOI:** 10.1101/gr.277698.123

**Published:** 2023-06

**Authors:** Jack A. Collora, Ya-Chi Ho

**Affiliations:** Department of Microbial Pathogenesis, Yale University School of Medicine, New Haven, Connecticut 06519, USA

## Abstract

HIV-1 integration introduces ectopic transcription factor binding sites into host chromatin. We postulate that the integrated provirus serves as an ectopic enhancer that recruits additional transcription factors to the integration locus, increases chromatin accessibility, changes 3D chromatin interactions, and enhances both retroviral and host gene expression. We used four well-characterized HIV-1-infected cell line clones having unique integration sites and low to high levels of HIV-1 expression. Using single-cell DOGMA-seq, which captured the heterogeneity of HIV-1 expression and host chromatin accessibility, we found that HIV-1 transcription correlated with HIV-1 accessibility and host chromatin accessibility. HIV-1 integration increased local host chromatin accessibility within an ∼5- to 30-kb distance. CRISPRa- and CRISPRi-mediated HIV-1 promoter activation and inhibition confirmed integration site–dependent HIV-1-driven changes of host chromatin accessibility. HIV-1 did not drive chromatin confirmation changes at the genomic level (by Hi-C) or the enhancer connectome (by H3K27ac HiChIP). Using 4C-seq to interrogate HIV-1–chromatin interactions, we found that HIV-1 interacted with host chromatin ∼100–300 kb from the integration site. By identifying chromatin regions having both increased transcription factor activity (by ATAC-seq) and HIV-1–chromatin interaction (by 4C-seq), we identified enrichment of ETS, RUNT, and ZNF-family transcription factor binding that may mediate HIV-1–host chromatin interactions. Our study has found that HIV-1 promoter activity increases host chromatin accessibility, and HIV-1 interacted with host chromatin within the existing chromatin boundaries in an integration site–dependent manner.

Three-dimensional (3D) spatial chromatin organization brings remote enhancers in contact with promoters and regulates gene expression in *cis*, allowing transcription factor binding and recruitment of coactivators, corepressors, and RNA polymerase. Chromatin structural proteins, such as CCCTC-binding factor (CTCF) and cohesion, form insulating boundaries and facilitate chromatin interactions within the same topological associating domains (TADs) and chromatin loops. As retroviruses integrate into the human genome, we postulate that the integrated provirus serves as an ectopic enhancer that recruits additional transcription factors to the integration locus, increases chromatin accessibility, alters 3D chromatin interactions, and enhances both retroviral and host gene expression. We wonder whether retrovirus-induced ectopic chromatin interactions can extend beyond chromatin boundaries or are restricted locally.

In human chromatin, existing retroviral promoter long terminal repeat (LTR) elements provide transcription factor binding sites that regulate gene expression across development, tissues, and cell types (Dunn et al. 2003; Bourque et al. 2008; Lamprecht et al. 2010; Thurman et al. 2012; Wang et al. 2012; Chuong et al. 2013, 2017; Jiang et al. 2014; Sundaram et al. 2014; Rayan et al. 2016; Thompson et al. 2016; Trizzino et al. 2017; Lee et al. 2020). These LTR elements may act as enhancers that recruit host transcription factors and induce chromatin loops to regulate gene expression across large genomic distances. For instance, the retroviral elements in the MER41B family have been co-opted for the interferon response (Chuong et al. 2016). In addition to these preexisting genomic elements, a modern human retrovirus, human T cell leukemia virus type 1 (HTLV-1), encodes an ectopic CTCF binding site (Satou et al. 2016). This ectopic insertion results in characteristic CTCF epigenetic insulator activity and induces novel genomic loops that alter the expression of HTLV-1 and the surrounding host genes (Melamed et al. 2018).

Although antiretroviral therapy (ART) stops new rounds of HIV-1 infection, the HIV-1 reservoir is established early during infection and persists lifelong (Chun et al. 1997; Finzi et al. 1997; Wong et al. 1997). In HIV-1^+^ individuals under suppressive ART, HIV-1 integration sites are enriched in cancer genes (Maldarelli et al. 2014; Wagner et al. 2014). HIV-1 integration into cancer genes drives aberrant cancer gene expression by HIV-1–host aberrant splicing (Liu et al. 2020), causing aberrant proliferation of the infected cells (Maldarelli et al. 2014; Wagner et al. 2014; Yoon et al. 2020) and even cancer transformation in rare cases (Mellors et al. 2021). On the other hand, HIV-1 integration into zinc-finger protein (ZNF) genes provides long-term persistence and a survival benefit for the infected cell in people treated with long-term ART (Huang et al. 2021; Einkauf et al. 2022) and in people who have natural control of HIV-1 infection (Jiang et al. 2020), presumably by reducing HIV-1 reactivation in the repressive chromatin environment near the ZNF genes. Understanding HIV-1–chromatin interactions at the integration site provides insights into the mechanisms of HIV-1 insertional mutagenesis and HIV-1 integration site–dependent proliferation of the infected cells and informs therapeutic interventions that can stop the proliferation of HIV-1-infected cells.

HIV-1 can induce chromatin looping in a transcription-dependent manner (Perkins et al. 2008). HIV-1 proviral genome harbors *cis*-regulatory elements that recruit transcription factors. HIV-1 proviral accessibility has been well characterized, with three regions of DNase I hypersensitivity on the USF1 and NF-kB binding sites in the LTR and within the coding sequence, which are remodeled upon transcriptional activation (Verdin 1991). Several key transcription factors, including NFATC1, NFATC2 (Romanchikova et al. 2003), and AP-1 (Roebuck et al. 1996), are known to bind the HIV-1 LTR. BCL11B (Cismasiu et al. 2008; Hu et al. 2018) and YY1 (Margolis et al. 1994; Weintraub et al. 2017) have been shown to interact with the HIV-1 LTR through intermediary proteins and are associated with 3D chromatin looping. We postulate that HIV-1 increases host gene expression beyond the transcription unit by increasing host chromatin accessibility, increasing enhancer activity, and altering 3D chromatin conformation.

Although the HIV-1 latent reservoir has been the major focus of research for HIV-1 eradication and cure, one major conceptual advancement in the field is to understand that transcriptionally active (as opposed to latent) HIV-1 proviruses can persist despite effective ART and can contribute to viral rebound during treatment interruptions (Collora and Ho 2022; Einkauf et al. 2022). Here, we used four well-characterized HIV-1-infected Jurkat T cell line clones having unique integration sites and low to high levels of HIV-1 expression to interrogate whether and how HIV-1 integration changes host chromatin accessibility (by single-cell DOGMA-seq), enhancer activity (by H3K27ac HiChIP), and 3D chromatin confirmation (by Hi-C). CRISPR activation (CRISPRa)– and CRISPR inactivation (CRISPRi)–mediated HIV-1 promoter activation and inhibition showed HIV-1-driven changes of host chromatin accessibility. Specifically, we used 4C-seq to capture HIV-1–host chromatin interactions and identified transcription factor binding motifs that mediate increased host chromatin accessibility and HIV-1–host chromatin interactions.

## Results

To examine HIV-1–host chromatin interactions, we used four established HIV-1-infected Jurkat T cell line clones (Liu et al. 2020; Yeh et al. 2020; Pedersen et al. 2022). HIV-1 proviruses in these cell lines are integrated into unique and known integration sites in introns of actively transcribed genes (Table 1), similar to HIV-1 integration sites in vivo in HIV-1^+^ individuals under suppressive ART (Schröder et al. 2002; Han et al. 2004; Ho et al. 2013). HIV-1 proviruses integrated in the same orientation as the host gene in these cell line clones drive high levels of aberrant host gene transcription through aberrant HIV-1-to-host RNA splicing as seen in vivo (Liu et al. 2020). Using four cell line clones as biological replicates enables us to interrogate how different HIV-1 integration sites impact HIV-1–host chromatin interactions.

**Table 1.**
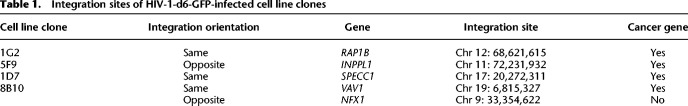
Integration sites of HIV-1-d6-GFP-infected cell line clones

### HIV-1 proviral chromatin accessibility correlates with host chromatin accessibility and HIV-1 RNA expression

**Figure 1.**
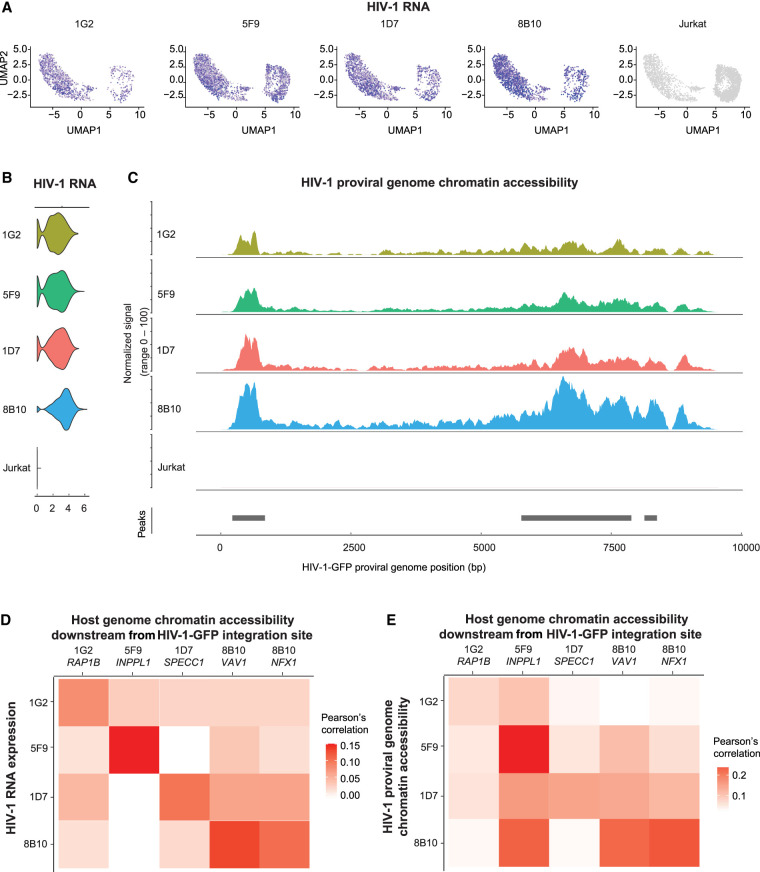
HIV-1 RNA expression correlates with HIV-1 proviral genome chromatin accessibility and host chromatin accessibility captured by single-cell DOGMA-seq. (*A*) UMAP dimensional reduction heatmap of HIV-1 RNA expression levels at the single-cell level in each HIV-1-infected cell line clone. (*B*) Log-normalized HIV-1 RNA expression level for each HIV-1-infected cell line clone and uninfected Jurkat clones. (*C*) Chromatin accessibility at the HIV-1 proviral genome across cell lines normalized to the total number of cells in each group by Signac. Peaks were identified by MACS2. (*D*) Pearson's correlations between HIV-1 RNA expression levels and host chromatin accessibility downstream from HIV-1 integration site across all cells within that clone. (*E*) Pearson's correlations between HIV-1 genome chromatin accessibility and host chromatin accessibility downstream from HIV-1 integration site across all cells within that clone. N = 11,333 cells total; 1314, 2869, 1853, 1906, and 3391 for 1G2, 5F9, 1D7, 8B10, and uninfected Jurkat cells, respectively.

**Figure 2.**
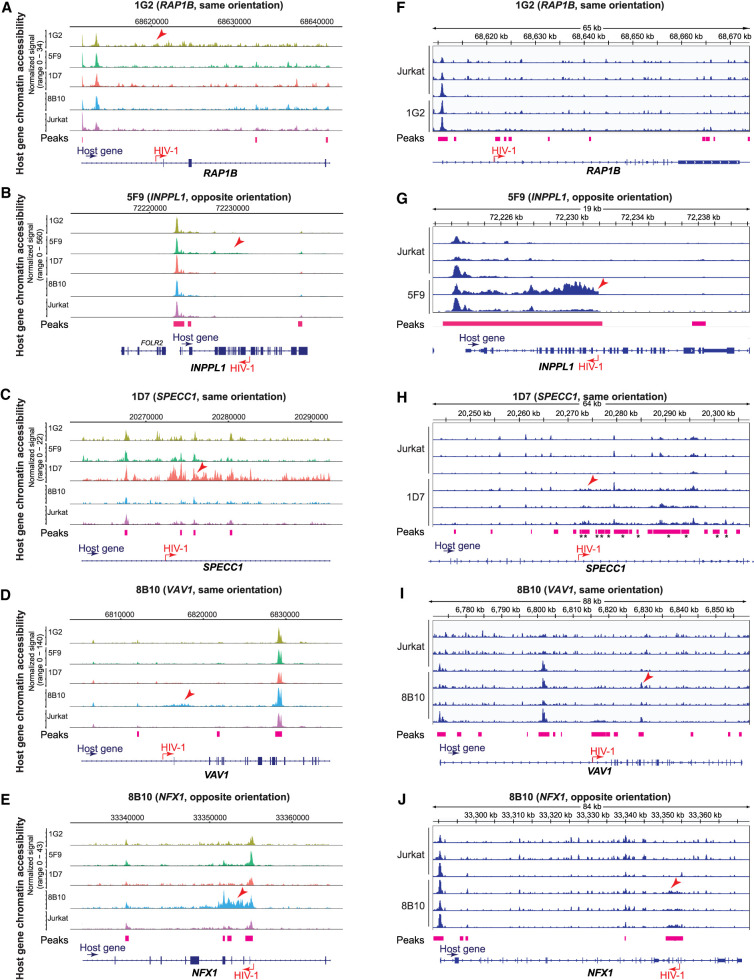
HIV-1 increases host chromatin accessibility ∼5–30 kb around the HIV-1 integration site. (*A*–*E*) Host chromatin accessibility captured by single-cell DOGMA-seq. (*F*–*J*) Host chromatin accessibility captured by bulk ATAC-seq. (*A*–*E*) Normalized by Signac. (*F*–*J*) Normalized by counts per million (CPM) and displaying a data range of zero to one. An asterisk denotes adjusted *P-*values < 0.05 by DESeq2 when comparing uninfected Jurkat T cells to the indicated HIV-1-infected clonal cell line. Red arrowheads indicate examples of increased host chromatin accessibility identified in respective cell line clones. Peaks shown were called by MACS2 for DOGMA-seq cells and by Genrich for bulk ATAC-seq.

HIV-1 gene expression is different in each cell because of the stochastic Tat feedback loop regulating HIV-1 transactivation (Weinberger et al. 2005). Therefore, although all four HIV-1-Jurkat cell clones carry an HIV-1 provirus in each cell, HIV-1 expression level is not 100%. To capture the heterogeneity of HIV-1 expression and its impact on chromatin accessibility, we profiled HIV-1 expression and chromatin accessibility at the single-cell level. We used DOGMA-seq (Mimitou et al. 2021) for paired single-cell RNA-seq and single-cell ATAC-seq to examine HIV-1 RNA expression, chromatin accessibility at the HIV-1 proviral genome, and chromatin accessibility at the host genome at the HIV-1 integration site within the same single cell. Different from paired single-nucleus RNA-seq and single-nucleus ATAC-seq, which captures RNA in the nucleus but not the cytoplasm, DOGMA-seq captures HIV-1 and cellular RNA in both the nucleus and the cytoplasm. First, we found that the HIV-1 RNA expression level was heterogeneous even within cells from the same clone despite having the same HIV-1 integration site, confirming the influence of the Tat feedback loop on the stochastic HIV-1 expression (Fig. 1A). The average HIV-1 RNA expression level differed in the four cell line clones (from low to high: 1G2, 5F9, 1D7, 8B10) (Fig. 1B). The chromatin accessibility at the HIV-1 genome differed (from low to high: 1G2, 5F9, 1D7, 8B10) (Fig. 1C), following the same trend as HIV-1 RNA expression. Second, when we examined correlations between HIV-1 RNA transcription, HIV-1 proviral genome chromatin accessibility, and host chromatin accessibility at the HIV-1 integration site, we found that HIV-1 RNA transcription correlated with HIV-1 proviral genome chromatin accessibility (Supplemental Fig. S1), HIV-1 RNA transcription correlated with the host chromatin accessibility downstream from the integration site (Fig. 1D), and HIV-1 proviral genome chromatin accessibility correlated with host genome chromatin accessibility downstream from the integration site (Fig. 1E). Third, the chromatin accessibility of the host genome downstream from the HIV-1 integration site increased, specifically at the HIV-1 integration site for each clone (Fig. 2A–E, red arrow heads). We confirmed the results using bulk ATAC-seq in replicates (Fig. 2F–J). Overall, we found that HIV-1 RNA expression, HIV-1 chromatin accessibility, and host chromatin accessibility at the integration site were strongly correlated.

### HIV-1 promoter activation increases host chromatin accessibility

**Figure 3.**
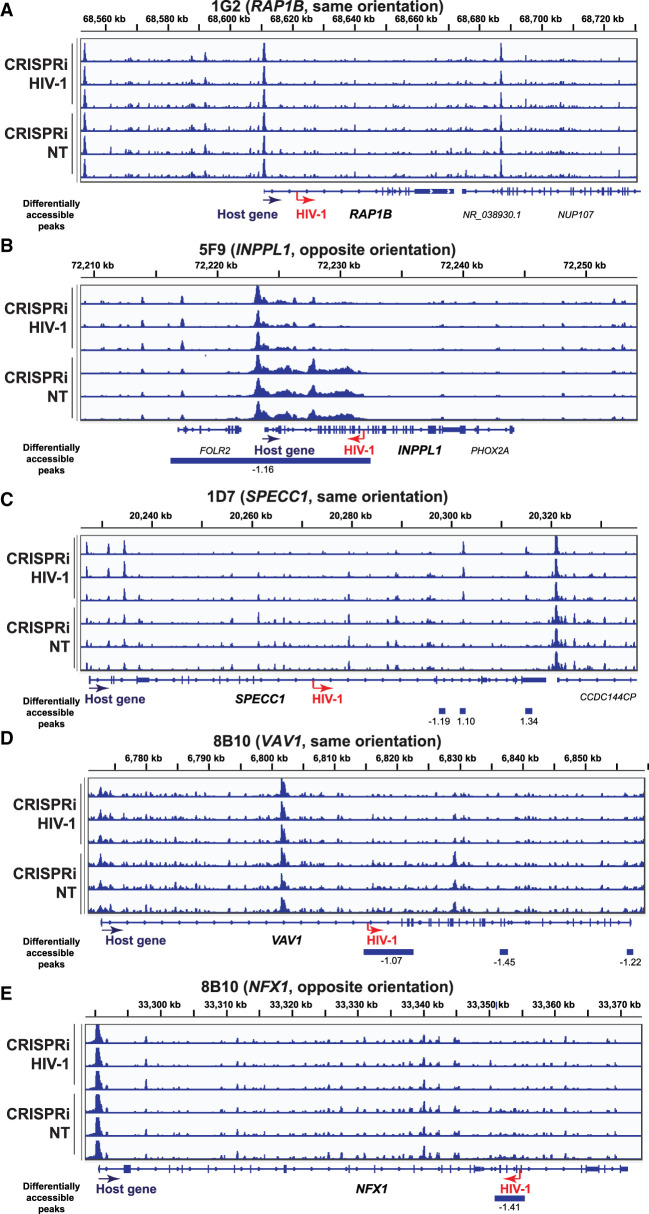
HIV-1 promoter activity repression reduces host chromatin accessibility near the HIV-1 integration site. (*A*–*E*) CRISPRi-mediated HIV-1 promoter inhibition reduces host chromatin accessibility near the integration site. ATAC-seq tracks were normalized by counts per million (CPM) scaled by the fraction of reads in peaks and display a data range of zero to three. Each peak is annotated with its log_2_ fold change relative to the nontargeting control. HIV-1 sgRNA targets HXB2 coordinates 344–363 in HIV-1 LTR promoter. (NT) Nontargeting gRNA. Statistics performed by DESeq2; all peaks displayed have an adjusted *P*-value < 0.05.

We noted that the degree of chromatin accessibility (Fig. 2) corresponded to the amount of GFP expression in our cell line clones (Supplemental Fig. S2). To understand the cause-effect relationship between HIV-1 and the observed increase in downstream chromatin accessibility, we wanted to examine whether HIV-1 promoter activity drove host genome chromatin accessibility downstream from the HIV-1 integration site. To examine the impact of HIV-1 promoter activity on host chromatin accessibility downstream from the integration site, we used sgRNA targeting HIV-1 promoter versus nontargeting controls (NT) with CRISPRa and CRISPRi cell line clones. We examined whether activation of the HIV promoter increased host chromatin accessibility downstream from the HIV-1 integration site and whether inhibiting the HIV-1 promoter decreased host chromatin accessibility downstream from the HIV-1 integration site. We have previously shown that CRISPRa-mediated HIV-1 promoter activation increases HIV-1-driven aberrant host gene expression at the integration site, whereas CRISPRi-mediated HIV-1 promoter inhibition suppresses HIV-1-driven host gene expression at the integration site (Liu et al. 2020). In examining additional integration sites, we found heterogeneity in chromatin responses to CRISPRa-mediated HIV-1 promoter activation and CRISPRi-mediated HIV-1 promoter inhibition. CRISPRi-mediated HIV-1 promoter inhibition significantly decreased chromatin accessibility at three out of the five integration sites (Fig. 3A–E). CRISPRa-mediated HIV-1 promoter activation increased chromatin accessibility at only one out of the five integration sites (Supplemental Fig. S3A–E). Of note, because host chromatin is already highly accessible at these HIV-1 integration sites (Fig. 2), further CRISPRa-mediated HIV-1 promoter activation did not increase chromatin accessibility relative to CRISPRa-NT (Supplemental Fig. S4A,B). These results suggest that HIV-1 promoter activity can remodel the host chromatin environment.

### Transcription factor activities differ in cell line clones having different HIV-1 integration sites

We wanted to identify transcription factor activity that is associated with HIV-1 transcription and increased chromatin accessibility. Presumably, the activity of transcription factors known to bind to HIV-1 promoter, such as NF-kB, NFATC1, and SP1, would be associated with increased chromatin accessibility at the integration site and HIV-1 RNA expression. When we examined chromatin accessibility and transcription factor binding at the bulk ATAC-seq level, we did not find prominent transcription factor motif enrichment in each individual cell line. Using chromVAR on the ATAC-seq profile from DOGMA-seq at the single-cell level, we first examined the transcription factors that are differentially active across cell lines (Supplemental Fig. S5A; Supplemental Table S1). We next asked whether these transcription factor activities correlate with HIV-1 chromatin accessibility. We found that transcription factor activities that correlated with HIV-1 chromatin accessibility (Supplemental Fig. S5B) were distinct from those that were differentially active across cell lines (Supplemental Fig. S5C).

We found that for transcription factors known to bind to HIV-1 promoters, such as NFATC1, NFATC2, and SP1, the activity of these transcription factors correlated with HIV-1 chromatin accessibility (Supplemental Fig. S5B). For transcription factors not known to bind to HIV-1, such as MEF2B, MEF2D, PDX1, and YY2, the activity of these transcription factors also correlated with HIV-1 chromatin accessibility (Supplemental Fig. S5B). Given that these cell lines harbored the same HIV-1 proviral genome having the same transcription factor binding sites, our results suggest that the difference in transcription factor activity association is related to different HIV-1 integration sites. Thus, the regulation of HIV-1 transcription and chromatin accessibility likely involves local elements, such as host enhancers and promoters regulated by the transcription factors at that site. Chromatin accessibility and transcription factor binding motif enrichment are not different between HIV-1-infected cell line clones and the uninfected Jurkat clone. Thus, regulation of HIV-1 transcription activity and chromatin accessibility depends on local *cis*-regulatory elements rather than the whole-cell state.

### HIV-1 modestly disrupts local enhancer–promoter interactions without impacting global chromatin structure

**Figure 4.**
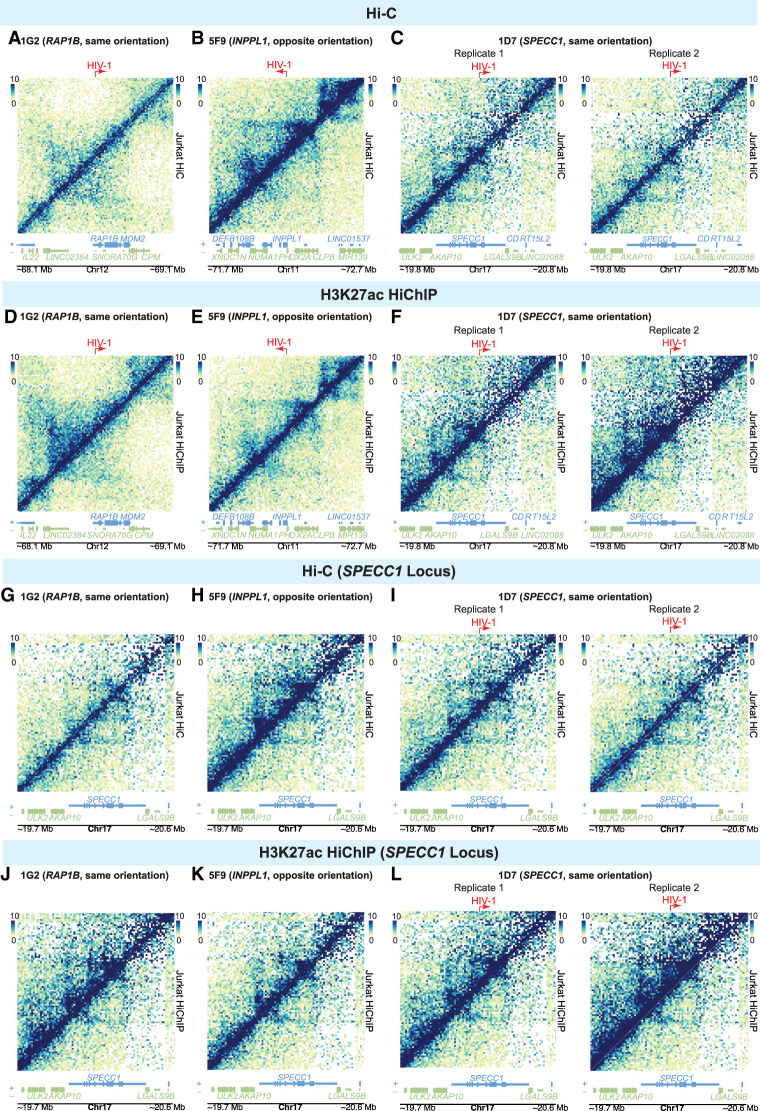
Impact of HIV-1 on genome-wide 3D chromatin conformation and H3K27ac enhancer loops at the integration site. Heatmap shows Knight–Ruiz (KR) normalized signal at 10-kb resolution surrounding their respective integration sites in Hi-C (*A*–*C*) and H3K27ac HiChIP (*D*–*F*). Heatmaps showing the Hi-C (*G*–*I*) and interaction (*J*–*L*) profiles at the same genomic window in lines with no HIV-1 integration into *SPECC1* (*G*,*H*,*J*,*K*) and those with HIV-1 integrated into *SPECC1* (*I*,*L*).

Long-range chromatin interactions regulate gene expression. We postulate that the HIV-1 proviral genome interacts with the host chromatin, creating integration site–dependent differences in transcriptional activity. We explored three 3D chromatin conformation capture approaches to interrogate HIV-1–host chromatin interactions and examined global chromatin interactions (by Hi-C) (Fig. 4A–C; Belton et al. 2012), enhancer-focused chromatin interactions (by H3K27ac HiChIP) (Fig. 4D–F; Mumbach et al. 2017), and HIV-1-focused interactions (by 4C-seq) (Fig. 6). Hi-C enables a nonbiased interrogation of genome-wide 3D chromatin structure and detects TADs that restrict interactions across the genome (Belton et al. 2012; Nora et al. 2012). Using Hi-C on our HIV-1-infected cell line clones, we did not observe a consistent significant impact of HIV-1 provirus on chromatin structure at the chromatin scale (Fig. 4A–C). To focus on chromatin interactions involving active enhancers and promoters, we used H3K27ac HiChIP (Mumbach et al. 2017). Briefly, we immunoprecipitated (IP) the active histone marker H3K27ac to examine chromatin interactions that are bound by H3K27ac. H3K27ac is a histone modification that marks active enhancers and promoters (Creyghton et al. 2010; Rada-Iglesias et al. 2011). We initially observed qualitative increases in chromatin interactions around HIV-1 integration sites as measured by H3K27ac HiChIP (Fig. 4D–F) relative to uninfected controls. When we further investigated whether this impact was an HIV-1-integration site–specific phenomenon or an HIV-1-infection–specific phenomenon, we found that our observed differences in Hi-C and H3K27ac HiChIP, when comparing infected and uninfected cells, were present in all HIV-1-infected lines regardless of whether those cells contained an integration site at that location (Fig. 4G–L) or were at model loci on separate chromosomes (for *BACH2*, see Supplemental Fig. S6A–F; for *MYC*, see Supplemental Fig. S6G–L). These changes were present despite similar quality-control metrics (Supplemental Table S2) across cell lines, suggesting the presence of HIV-1 may nonspecifically enhance chromatin interactions.

To further analyze these data and make statistical comparisons, we used a pairwise normalization and comparison approach in HiCcompare (Stansfield et al. 2018). In contrast to the typical Knight–Ruiz (KR) normalization applied to genome-wide chromatin interaction data, HiCcompare implements Loess normalization and comparison of those normalized values, which is more robust to correct technical variance than KR normalization. We observed differences in signal across comparisons, but there were no interaction domains that were consistently, statically, and significantly up-regulated or down-regulated in HIV-1-infected cells relative to each other or relative to uninfected controls (Supplemental Table S3). Direct HIV-1–host chromatin interactions were sparse. Overall, our results suggest that HIV-1 has a minimal impact on local enhancer–promoter interactions and on global chromatin structure.

### HIV-1 interacts with host chromatin in *cis* at the integration site

We postulate that the impact of the HIV-1 proviral genome on chromatin structure is at the HIV-1 integration site locally. Circularized chromatin conformation capture sequencing (4C-seq) (Zhao et al. 2006) enables capturing local chromatin interactions at a specific genome region of interest and enhances resolution locally compared with Hi-C and HiChIP. Briefly, each cell line clone was cross-linked by paraformaldehyde to lock interacting chromatins in physical contact. Chromatin was digested by a restriction enzyme NlaIII and ligated by T4 ligase. NlaIII recognizes and digests 4 bp of DNA (CATG), creating a high-resolution digestion of the human genome (an average of one cut per 4^4^ = 256 bp). Therefore, distal chromatin regions in close physical contact were ligated to form chimeric DNA regions. After reverse cross-linking, the DNA fragments were further digested by another restriction enzyme, MluCl (cutting AATT), into smaller fragments and circularized by intramolecular ligation in dilute conditions. These circularized chromatin contacts were amplified by HIV-1-specific primers targeting the HIV-1 core promoter and enhancer (HXB2 coordinates 249–728), where most transcription factors bind. Of note, the two restriction enzymes NlaIII and MluCI were carefully chosen because they preserve a large portion of the HIV-1 promoter region of interest in NL4-3-d6-dsGFP reporter virus in the cell line clones. To quantify chromatin interactions, we used the percentage of possible fragments detected (as opposed to direct read numbers) because the restriction enzyme digestion method, as opposed to shearing-based methods, lacks a unique molecular identifier (UMI) (van de Werken et al. 2012). Specifically, we first determined all fragments that were interacting between HIV-1 and the human genome for each sample. We adopted a strategy similar to previous approaches (Melamed et al. 2018). A threshold of 10 reads was defined as the presence of HIV-1–human genome interactions. Next, we counted the number of fragments captured in a 40-restriction-fragment window (one restriction fragment is on average 4^4^ = 256 bp in length; the average distance of 40 consecutive 256-bp fragments spans ∼256 bp per fragment × 40 fragments = 10,240 bp) tiled in increments of five fragments (256 bp per fragment × five fragments = 1280 bp on average) across the whole genome. The capture of multiple independent fragments within a given region allows us to approximate the interaction likelihood without relying on read count. We then plotted that number across the genome.

**Figure 5.**
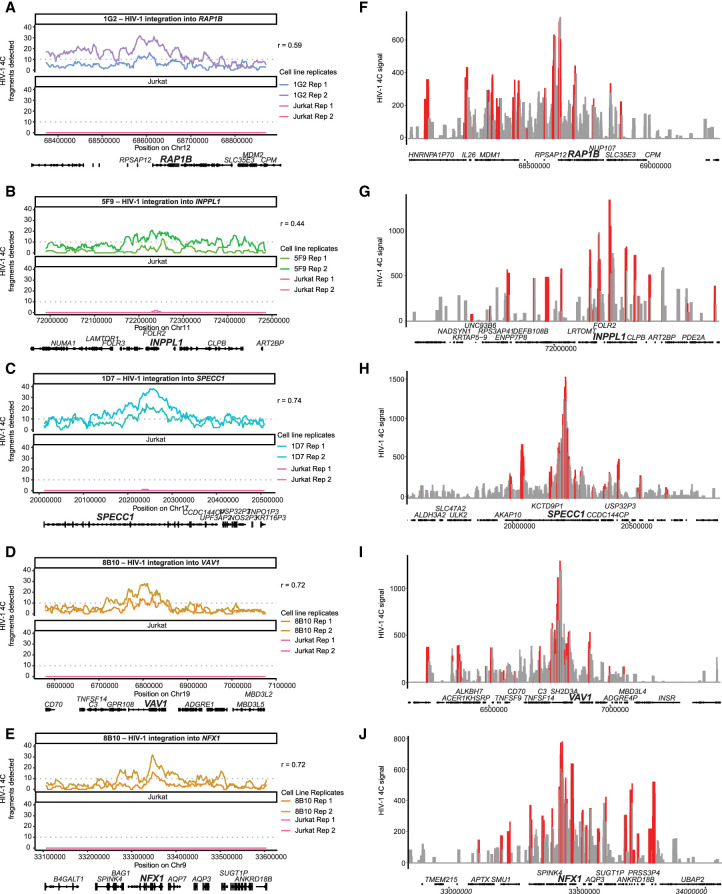
HIV-1–host chromatin interactions captured by HIV-1 4C-seq. (*A*–*E*) Genomic tracks of the number of fragments detected (captured with at least 10 reads) in a running 40-fragment window, which was slid five fragments at a time. Shown is a 500-kb window centered on the integration site. Signals in Jurkat 4C-seq libraries were shown for comparison. Pearson correlation coefficient between experimental replicates is indicated. Dotted line indicates 10 fragments, the threshold chosen for overlapping with ATAC-seq peaks. (*F*–*J*) Genomic tracks of the read normalized signal as calculated by peakC in a 21-fragment sliding window in a 1-Mb region centered on the integration site. Red regions indicate *P* < 0.05 relative to distance-based model.

Despite differences in the magnitude of signal between replicates, we observed a correlation between each of our replicates (Pearson's correlation 0.44–0.74) (Fig. 5A–E). Uninfected Jurkat T cells had no significant interactions at HIV-1 integration sites in other cell line clones, suggesting the amplification was specific for HIV-1 interactions. In each cell line clone, we found HIV-1–host chromatin interactions ∼100 kb away (Fig. 5A–E). We did not detect significant interactions (more than 10 restriction fragments out of 40 possible in a genomic window) on any chromosome other than those that contained the integration site itself. Our results indicate that the HIV-1 proviral genome does interact with surrounding chromatin.

Although only semiquantitative in nature, 4C-seq signal is expected to decay with distance from the region of interest. This property can be used to fit a monotonic model, which the observed data can then be tested against (Geeven et al. 2018). This approach is more sensitive to interactions because it models the expected decrease in signal to detect weaker long-range interactions. We used this method to examine individual integration sites across multiple replicates to understand if there were lower magnitude distal regions that HIV-1 may interact with. We identified significant HIV-1–host chromatin interactions in all four cell line clones (up to 100 kb–300 kb) both upstream of and downstream from the HIV-1 integration site (Fig. 5F–J, in red). We found that the HIV-1 genome interacted more with the upstream host chromatin than the downstream chromatin, particularly when HIV-1 is integrated in the same orientation as the host transcription unit (Fig. 5F,H,I). Overall, we found that HIV-1 interacts with chromatin ∼100 kb–300 kb upstream of and downstream from the HIV-1 integration site.

### The HIV-1 LTR interacts with accessible chromatin enriched in binding motifs of host transcription factors, including ZNF proteins and ETS and RUNX transcription factor families

**Figure 6.**
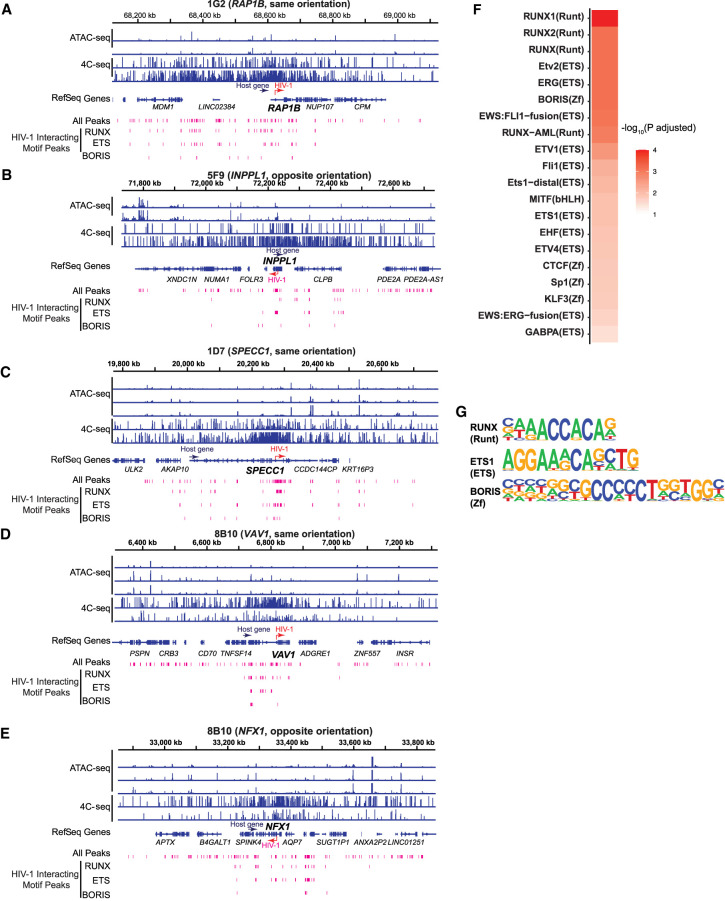
Transcription factor binding motifs enriched in both increased chromatin accessibility and HIV-1–host chromatin interactions. (*A*–*E*) HIV-1 interacting peaks, defined as regions of overlapping ATAC-seq peaks and 4C-seq signal (fragments detected were 10 or more in at least one replicate out of 40 possible), were extracted from the integrated chromosome in each cell line. (*F*) Transcription factor binding motifs that were enriched in HIV-1 interacting peaks were identified by HOMER. Top 20 known motifs ranked by adjusted *P-*values. (*G*) Example motif logos for each of the top three transcription factor groups: ETS, RUNX, and ZNF. *P*-values were corrected for multiple hypothesis testing by the Benjamini–Hochberg procedure.

We postulate that transcription factors mediate chromatin binding, looping, and 3D interactions between HIV-1 promoter and local host chromatin. We compared ATAC-seq peaks and 4C-seq at the integration site to identify transcription factors mediating local HIV-1–chromatin interactions. We identified genome regions having both increased chromatin accessibility (from ATAC-seq) and increased chromatin interactions (from 4C-seq), focusing on ATAC-seq peaks (size <1 kb) around the HIV-1 integration site that overlapped windows of HIV-1–host chromatin interaction as determined by 4C-seq (size ∼10 kb) (Fig. 6A–E). Each cell line had few interacting peaks (66 from 1G2, 30 from 5F9, 53 from 1D7, and 55 from 8B10), which resulted in relatively few poorly enriched motifs at any one site (Supplemental Fig. S7). To identify host factors that may commonly underlie HIV-1–host chromatin interactions, we examined regions of HIV-1–host chromatin interactions from all five integration sites. We used HOMER (Heinz et al. 2010) to search for transcription factor binding motif enrichment in the selected regions relative to the host background.

We found several host transcription factor binding motifs in these regions of interaction (Fig. 6F). Several factors within the same family were repeatedly identified in the top 20 enriched motifs, including 11 ETS motifs, four RUNX motifs, and four zinc-finger (ZF) motifs (Fig. 6G). Of note, members of the ETS and RUNX transcription factor families have been reported to bind the HIV-1 LTR as well (Sieweke et al. 1998; Hultquist et al. 2012; Bosque et al. 2017). Additionally, we identified enrichment of ZF protein binding motifs that were associated with both increased chromatin accessibility and increased HIV-1–host chromatin interactions at the HIV-1 integration site, including ZF transcription factors known to regulate HIV transcription, such as CTCFL (encoded by *CTCFL*, a paralog of CTCF) (Jefferys et al. 2021), ZFX (known to induce HIV-1 transactivation) (Gazin 1999), PLAGL1 (Pedro et al. 2021), and KLF3 (Pedro et al. 2021). *ZNF652* is a known frequent HIV-1 integration site in HIV-1^+^ individuals (Maldarelli et al. 2014; Wagner et al. 2014). Recent studies have identified that HIV-1 integration into ZF protein genes provides a survival benefit for the infected cells (Jiang et al. 2020; Huang et al. 2021). Our study identified transcription factors that may regulate HIV-1 chromatin accessibility and HIV-1–host interactions at the integration site.

## Discussion

Viral infection, such as influenza and SARS-CoV-2, can disrupt chromatin structure independently of host factors binding to viral genomes, through both aberrant transcription and cohesion depletion (Heinz et al. 2018; Wang et al. 2023). Given that our reporter virus has inactivation mutations in most viral proteins (Gag, Pol, Vif, Vpr, Vpu, Env, and Nef), the effect of HIV-1–host chromatin interactions is likely mediated by *cis*-regulatory elements (such as transcription factor binding sites) on the HIV-1 genome, not mediated by viral proteins. Unlike HTLV-1, which has a CTCF binding site in the proviral genome, which changes chromatin looping, HIV-1 does not contain CTCF binding sites in the proviral genome (Satou et al. 2016). The impact of HIV-1 on host gene expression is thought to be limited within the transcription unit (Liu et al. 2020). Using RNA-seq, we have previously shown that HIV-1 promoter activity dominates over host gene promoter activity and drives high levels of aberrant host gene expression (Liu et al. 2020). This study found that HIV-1 not only increases host gene transcription but also increases host chromatin accessibility and may interact with surrounding chromatin.

This study suggests HIV-1 chromatin accessibility is driven by HIV-1 promoter activity in a context-dependent manner. CRISPRa-mediated HIV-1 activation failed to increase chromatin accessibility, whereas CRISPRi was able to suppress HIV-1-associated chromatin accessibility. This may be because of the high basal HIV-1 transcriptional activity of these cell lines, leading to a maximal HIV-1 expression without further activation. Additionally, the suppressible nature of this chromatin accessibility suggests that HIV-1-mediated chromatin changes are reversible. Furthermore, 4C-seq showed that HIV-1 induces chromatin looping between HIV-1 and host chromatin up to 100–300 kb away from the HIV-1 integration site, indicating that the impact of HIV-1 on host gene expression can extend beyond the transcription unit into adjacent genes. These interactions also preferred interacting upstream of integration, suggesting models of HIV-1–host chromatin determinants may be improved by weighing upstream factors more highly than downstream factors. This is the first study showing that the HIV-1 proviral genome interacts with host chromatin in *cis*.

By integrating HIV-1-interacting regions (by 4C-seq) and transcription factor binding motifs (by ATAC-seq), we identified transcription factors that may mediate HIV-1–host chromatin looping, such as ZF transcription factors and ETS and RUNX family transcription factors. These factors are of great significance as many of them are known to bind HIV-1 and enhance or suppress HIV-1 transcription. Furthermore, recent studies have found an enrichment for HIV-1 integrations into ZNF genes after long-term suppressive therapy (Jiang et al. 2020; Huang et al. 2021; Lian et al. 2023). Although integration into a given gene does not promote binding of that gene product to the provirus, it is possible that this diverse group of transcription factors regulate themselves in a feedback loop, giving HIV-1 access to those factors for its own regulation. These genes are also targets of the HUSH complex, which will deposit inhibitory histone modifications, which may reduce chromatin accessibility and HIV-1 transcriptional activity (Tchasovnikarova et al. 2015). We did not see an enrichment for other factors that have been reported to bind the LTR such as LEF1 and AP-1 transcription factors (Sheridan et al. 1995; Duverger et al. 2013). We speculate that the transcription factors available for HIV-1–host chromatin interactions are dictated by the local host environment, suggesting that at different integration sites, different factors may drive the interactions between HIV-1 and host. This heterogeneity may help explain the unique reactivation profile of distinct integration sites as others have previously reported (Chen et al. 2017; Einkauf et al. 2022). Understanding the interplay between HIV-1 integration and the chromatin environment around HIV-1 is crucial for achieving an HIV-1 cure.

Despite consistent interaction profiles observed by targeted amplification (4C-seq), we were not able to detect statistically robust changes in chromatin conformation (by Hi-C) or enhancer–promoter interactions (by H3K27ac HiChIP) around the integration site. It is possible that HIV-1 promoter activity and interactions are subjugated to existing host architecture or that the tendency of HIV-1 to form tight transcription and splicing linked loops (Perkins et al. 2008) prevents HIV-1-driven chromatin reprogramming. Jurkat cells are hypotetraploid (Gioia et al. 2018), obscuring the impact of HIV-1 on the one infected chromosome versus three uninfected chromosomes in each cell, particularly in the context of genome-wide data sets such as ATAC-seq, Hi-C, and HiChIP. It is possible that this current study represents a minimum chromatin interaction profile and that further chromatin studies could be conducted in a method that can use single-nucleotide polymorphisms (SNPs) to differentiate different chromosome arms and enable differentiation of chromosomal interactions. Approaches such as single-cell Hi-C (Ramani et al. 2017) and single-cell CUT&Tag (Bartosovic et al. 2021) for specific transcription factors may allow future studies to examine these changes directly and capture the heterogeneity of HIV-1–chromatin interactions across cells and in many integration sites. These single-cell approaches may build a sufficiently large number of chromatin profiles to perform robust statistical measures of the impact and heterogeneity of HIV-1–host chromatin interactions.

Although we chose cell line clones harboring HIV-1 integration into introns of actively transcribed genes and cancer genes to recapitulate HIV-1 integration events in HIV-1^+^ individuals, these integration sites serve as biological replicates but not as a precise reflection of HIV-1 integration into enriched integration sites, such as *BACH2*, *MRTFB*, and *STAT3* (Maldarelli et al. 2014; Yoon et al. 2020; Coffin et al. 2021; Mellors et al. 2021). To examine how HIV-1 promoter activity shapes HIV-1–chromatin interactions, we chose cell line clones that are transcriptionally active and do not reflect the latent state of HIV-1-infected cells. Further, recent studies have emphasized the changes in HIV-1 integration over long-term therapy (Einkauf et al. 2022) and in elite controllers (Jiang et al. 2020) toward transcriptionally repressive regions resistant to genomic study such as centromeres. It seems to be that in these scenarios, HIV-1 expression levels passively follow surrounding chromatin environment. This shift in integration sites reduces HIV-1 transcriptional activity, which our study suggests should result in less HIV-1-associated chromatin accessibility. Although we did not examine it directly, these results also suggest that its ability to make interactions with surrounding chromatin will also be reduced.

HIV-1 promoter activity increases host chromatin accessibility at the integration site in a transcription-dependent manner. 4C-seq identified HIV-1–host chromatin interactions up to 300 kb from the integration site. Specifically, we used 4C-seq to capture HIV-1–host chromatin interactions and identified transcription factor binding motifs that mediate increased host chromatin accessibility and HIV-1–host chromatin interactions.

## Methods

### Cell culture

HIV-1-d6-GFP-Jurkat T cell clones were generated by infecting Jurkat T cells as previously described (Liu et al. 2020; Yeh et al. 2020; Pedersen et al. 2022) at a low MOI with a single-round reporter virus HIV-1-d6-GFP (Yang et al. 2009). Three days after infection, GFP^+^ cells were sorted as one cell per well in 96-well plates and grown into clones. Four cell line clones—1G2 (harboring an HIV-1-d6-GFP provirus in *RAP1B*), 5F9 (harboring an HIV-1-d6-GFP provirus in *INPPL1*), 1D7 (harboring an HIV-1-d6-GFP provirus in *SPECC1*), and 8B10 (harboring two HIV-1-d6-GFP proviruses, one in *VAV1* and one in *NFX1*)—were selected for this study. HIV-1 integration sites were examined by inverse PCR (Han et al. 2004). Of these four clones, three—8B10, 1D7, and 1G2—were previously characterized (Liu et al. 2020; Yeh et al. 2020). Cell lines were cultured in RPMI 1640 (Thermo Fisher Scientific 11875093) supplemented with 10% heat-inactivated fetal bovine serum (Thermo Fisher Scientific 10438026). The Lenti-X 293T cell line (Takara Bio 632180) was cultured in DMEM (Thermo Fisher Scientific 11965118) supplemented with 10% heat-inactivated fetal bovine serum.

### Production of pseudotyped lentiviruses

Lenti-X 293T cells were seeded to obtain 70%–90% confluence the following day. The next day, cells were transfected using Lipofectamine 2000 transfection reagent (Thermo Fisher Scientific 11668019) with pMDLg/pRRE (Addgene 12251), pRSV-Rev (Addgene 12253), and pCMV-VSV-G (Addgene 8454) at an equal molecular ratio. Media was changed 6 h after transfection, and pseudotyped lentivirus was harvested 48–72 h after transfection. Viruses were concentrated using Lenti-X concentrator (Takara Bio 631231) or by ultracentrifugation using a 20% sucrose cushion.

### CRISPRi- and CRISPRa-ready cell generation, nontargeting, and HIV-1 targeting guides

For CRISPRi cell lines, Jurkat clones were transduced with pseudotyped lentiviruses encoding Lenti-dCas9-KRAB-blast (Addgene 89567) by spinoculation. Two days posttransduction, 8 µg/mL blasticidin was added to the culture medium to select transduced cells. After 1 wk of blasticidin selection, cells were transduced with pseudotyped viruses encoding gRNAs targeting the HIV-1 LTR (CTACAAGGGACTTTCCGCTG) or nontargeting (GTGCACCCGGCTAGGACCGG), cloned into the parental plasmid pCRISPRia-v2 (Addgene 84832). Two days after transduction, 1.5 µg/mL puromycin was added to the culture medium to select transduced cells.

For CRISPRa cell lines, Jurkat clones were transduced with pseudotyped lentiviruses encoding Lenti-MPH v2 (Addgene 89308) by spinoculation. Two days posttransduction, 600 µg/mL of hygromycin was added to the culture medium to select transduced cells. After 1 wk of hygromycin selection, cells were transduced with pseudotyped viruses encoding gRNAs targeting the HIV-1 LTR (CTACAAGGGACTTTCCGCTG) or nontargeting (GTGCACCCGGCTAGGACCGG), cloned into the parental plasmid lentiSAMv2 (Addgene 75112). Two days post-transduction, 1.5 µg/mL puromycin was added to the culture medium to select transduced cells. Cells were cultured for at least 7 d under selection before genomic assays.

### Flow cytometry

At least 2 million cells were harvested, pelleted, and washed once using wash media. Cells were then fixed by resuspending them in 4% paraformaldehyde. Cells were then analyzed on a FACSAria cell sorter. Gating and quantification were performed using FlowJo (BD Biosciences, version 10.8.0)

### DOGMA-seq

Cells were prepared for DOGMA-seq as previously described (Mimitou et al. 2021). Dead cells were removed using an EasySep dead cell removal kit (Stemcell Technologies 17899). The four HIV-1-d6-GFP-Jurkat T cell clones (1G2, 5F9, 1D7, and 8B10) were labeled using TotalSeq-A human hashing antibodies (1–4; BioLegend 394661, 394663, 394665, 394667) and pooled in equal numbers. Uninfected Jurkat cells and the pool of Jurkat clones were lysed on ice using DIG lysis buffer (20 mM Tris-HCl, 150 mM NaCl, 3 mM MgCl_2_, 0.01% digitonin, 2 U/µL RNase inhibitor) for 5 min before the addition of DIG wash buffer (20 mM Tris-HCl, 150 mM NaCl, 3 mM MgCl_2_, 1 U/µL RNase inhibitor). Cells were washed once with DIG wash buffer and then loaded onto the 10x Chromium controller (10x Genomics) for the single-cell multiome ATAC + Gene Expression assay according to the manufacturer's protocol (10x Genomics document CG000338).

Libraries were prepared according to the same protocol with the following modifications. During the barcoding reaction (step 4.1), 1 µL of 0.2 µM HTO oligo (GTGACTGGAGTTCAGACGTGTGC*T*C) was added. During the following purification (step 4.3.k), beads were eluted in 100 µL. A barcoding PCR was added to construct the index protein tags using 35 µL of the eluted sample, 2.5 µL of 10 µM SI-PCR, 2.5 µL of 10 µM D7xx, and 50 µL 2× KAPA mix (Roche KK2601). The reaction was run on a thermocycler starting with 3 min at 95°C; 12 cycles of 20 sec at 95°C, 30 sec at 60°C, and 20 sec at 72°C; with a final extension of 5 min at 72°C. Libraries for ATAC-seq, transcriptome, and surface protein tags were sequenced on a NovaSeq 6000 in manufacturer-suggested read configurations.

### ATAC-seq

ATAC-seq was performed as previously described (Corces et al. 2017). Briefly, 50,000 cells were lysed on ice for 3 min in 50 µL ATAC-lysis buffer (10 mM Tris-HCl at pH 7.4, 10 mM NaCl, 3 mM MgCl_2_, 0.1% Tween 20, 0.1% NP-40, 0.01% digitonin) before the addition of 1 mL of ATAC-wash buffer (10 mM Tris-HCl at pH 7.4, 10 mM NaCl, 3 mM MgCl_2_, 0.1% Tween-20). Cells were pelleted at 4°C and then transposed with ATAC-transposition mix (1× TD buffer with 33% PBS, 0.1% Tween-20, 0.01% digitonin, 2.5 µL TDE1) for 30 min at 37°C. DNA was isolated using a MinElute PCR cleanup kit (Qiagen 28004) and eluted in 10 µL twice. The resulting DNA was amplified using NEBNext ultra II Q5 master mix (New England Biolabs M0544X) and Nextera indexing primers for 5 min at 72°C and 1 min at 98°C; followed by five cycles of 15 sec at 98°C, 30 sec at 63°C, and 1 min at 72°C; with a final extension of 5 min at 72°C. Additional cycles were added based on qPCR measurements of library amplification for a total of eight to 20 cycles per library. Libraries were then sequenced on a NovaSeq 6000 in 150-bp paired-end mode.

### 4C-seq

Five million to 10 million cells were cross-linked in 2% formaldehyde for 10 min at room temperature with shaking. The reaction was quenched by adding glycine to a final concentration of 0.125 M and shaking for 10 min at room temperature. Cross-linked cells were washed with ice-cold PBS twice before being incubated in Hi-C lysis buffer (10 mM Tris-HCl at pH 7.4, 10 mM NaCl, 0.2% NP-40) on ice for 30 min with protease inhibitors (Sigma-Aldrich 11873580001). The resulting nuclei were washed once with Hi-C lysis buffer without protease inhibitors and then resuspended in 50 µL 0.5% SDS for 7 min at 62°C. To quench SDS, 145 µL of water and 25 µL 10% Triton X-100 were added, and samples were incubated with 500 rpm shaking for 15 min at 37°C. After SDS quenching, samples were incubated in restriction enzyme 1 mix (200 U NlaIII, 25 µL 10× CutSmart buffer; New England Biolabs B6004S) with 500 rpm shaking for 4 h at 37°C. Two hundred units of NlaIII was added, and nuclei were incubated with 500 rpm shaking overnight at 37°C. The following morning, nuclei were heated for 20 min to 62°C to inactivate the restriction enzyme. One thousand microliters of T4 ligase mix (1.2× T4 ligase buffer; New England Biolabs B0202S) and 2000 units T4 DNA ligase (New England Biolabs M0202S) were added, and nuclei were incubated with 500 rpm shaking for 6 h at 25°C. Nuclei were pelleted and resuspended in 300 µL reverse cross-linking mix (10 mM Tris HCl at pH 7.4, 1% SDS, 0.5 mM NaCl, 1000 units Proteinase K; New England Biolabs P8102). Nuclei were incubated for 1 h with 500 rpm shaking at 55°C, and then the temperature was raised to 68°C for overnight incubation.

The following day, DNA was isolated using a phenol–chloroform extraction and ethanol precipitation. All DNA was then incubated with 500 rpm shaking in 200 µL restriction enzyme mix 2 (1× CutSmart 200 U MluCI; New England Biolabs R0538L) for 4 h at 37°C. An additional 200 U of restriction enzyme was added, and the incubation continued overnight. DNA was ethanol precipitated and resuspended in 14 mL T4 sparse ligation mix (1× T4 DNA ligase buffer, 2000 units T4 DNA ligase) and incubated overnight at 16°C. The following day, DNA was ethanol precipitated. DNA was initially amplified using NEBNEXT ultra II Q5 master mix in eight 25-µL reactions with ∼1 µg DNA with a program of 2 min at 98°C; followed by 30 cycles of 10 sec at 98°C, 30 sec at 68°C, and 1 min at 72°C, reducing the annealing temperature by 1°C per cycle until 63°C was reached; with a final extension time of 5 min at 72°C. DNA was pooled across reactions and isolated using a PCR cleanup kit. Fifty nanograms of resulting PCR product was then barcoded using Nextera primers for 5 min at 72°C and 1 min at 98°C; followed by five cycles of 15 sec at 98°C, 30 sec at 63°C, and 1 min at 72°C; with a final extension for 5 min at 72°C. The resulting libraries were sequenced using a NovaSeq 6000 or HiSeq 4000 in 100-bp pair-end or 150-bp pair-end modes.

### Hi-C and HiChIP

Hi-C and HiChIP libraries were produced as previously described (Mumbach et al. 2016, 2017) with minor modifications. Five million cells were cross-linked with 1% freshly made formaldehyde in 1 mL. Cells were incubated with shaking for 10 min at room temperature. Glycine was then added to a concentration of 0.125 M. Cells were incubated at room temperature for 5 min. Cells were resuspended in 500 µL of ice-cold Hi-C lysis buffer (10 mM Tris-HCl at pH 7.4, 10 mM NaCl, 0.2% NP-40, 1× Roche protease inhibitors; Roche 11697498001) and incubated with rotation for 30 min at 4°C. Nuclei were pelleted at 2500 *g* for 5 min at 4°C and washed once with Hi-C lysis buffer. The supernatant was removed, and the pellet was resuspended in 100 µL 0.5% SDS and incubated for 10 min at 62°C without shaking or rotation. Then, 285 µL of water and 50 µL 10% Triton X-100 were added to the sample. The sample was incubated at 37°C with 500 rpm shaking for 15 min. Fifty microliters of 10× CutSmart buffer (New England Biolabs B6004S) and 375 U of MboI (New England Biolabs R0147M) were added before the sample was shaken at 500 rpm for 2 h at 37°C. Samples were incubated for 20 min at 62°C to inactivate the restriction enzyme without rotation. Ends were filled in and marked with biotin with rotation for 1 h at 37°C after the addition of biotin master mix (37.5 µL of 0.4 mM biotin-dATP; Thermo Fisher Scientific 19524016); 4.5 µL each of 10 mM dCTP, dGTP, dTTP; and 50 U of DNA Polymerase I, large (Klenow) fragment (New England Biolabs M0210). Nine hundred forty-eight microliters of ligation master mix was then added (150 µL of 10× NEB T4 DNA ligase buffer, 125 µL of 10% Triton X-100, 3 µL of 50 mg/mL: BSA [New England Biolabs B9001], 4000 units T4 DNA ligase, and 660 µL of water). The reaction was then incubated with rotation for 4 h at room temperature before pelleting at 2500*g* for 5 min at room temperature. The supernatant was removed.

Nuclei were pelleted and brought up to 880 µL in nuclear lysis buffer (50 mM Tris-HCl at pH 7.4, 10 mM EDTA, 1% SDS, 1× Roche protease inhibitors 11697498001) and transferred to a Covaris millitube (Covaris 520135). Samples were sheared on a Covaris S220 for 2 min with a fill level of 10, duty cycle of five, PIP of 140, and cycles/burst of 200. Sheared samples were clarified by centrifugation for 15 min at 16,000*g* at 4°C. Clarified supernatant was diluted with an equal volume of 2× ChIP dilution buffer (0.01% SDS, 1.1% Triton X-100, 1.2 mM EDTA, 16.7 mM Tris-HCl at pH 7.4, 167 mM NaCl) in a new tube.

For H3K27ac HiChIP only, the supernatant was then incubated with 60 µL of Protein A beads (New England Biolabs S1425S) for 1 h with rotation at 4°C. Samples were placed on a magnet, transferred to a new tube, and incubated with 7.5 µg of H3K27ac antibody (Abcam ab4729) with rotation overnight at 4°C. Sixty microliters of Protein A beads was added the following morning and incubated with rotation for 1 h at 4°C. Three washes each of low-salt wash buffer (0.1% SDS, 1% Triton X-100, 2 mM EDTA, 20 mM Tris-HCl at pH 7.4, 150 mM NaCl), high-salt wash buffer (0.1% SDS, 1% Triton X-100, 2 mM EDTA, 20 mM Tris-HCl at pH 7.4, 500 mM NaCl), and LiCl wash buffer (10 mM Tris-HCl at pH 7.4, 250 mM LiCl, 1% NP-40, 1% sodium deoxycholate, 1 mM EDTA) were performed at room temperature. Beads were eluted in 50 mM sodium bicarbonate (pH 8.0), 1% SDS for 10 min with rotation followed by 3 min at 37°C with shaking. Beads were placed on a magnet, and the supernatant was transferred to a new tube. Two total elusions were performed. The 300 µL of eluted sample was incubated with 15 µL of Proteinase K (New England Biolabs P8107S) with 500 rpm shaking for 45 min at 55°C to promote protein digestion. The temperature was increased to 67°C for 1.5 h to reverse cross-links, and samples were purified using a DNA clean and concentrator kit (Zymo Research D4003) and eluted in 10 µL of water.

For all samples, 5 µL of Streptavidin C-1 beads (Thermo Fisher Scientific 65001) were washed with Tween wash buffer (5 mM Tris-HCl at pH 7.4, 0.5 mM EDTA, 1 M NaCl, 0.05% Tween-20) and then resuspended in binding buffer (10 mM Tris-HCl at pH 7.4, 1 mM EDTA, 2 M NaCl). Beads were incubated for 15 min with shaking and then washed twice with Tween wash buffer. Beads were incubated for 2 min at 55°C with 500 rpm shaking and then washed with 100 µL of 1× TD buffer (10 mM Tris HCl at pH 7.5, 5 mM MgCl_2_, 30% dimethylformamide). The resulting beads were resuspended with 4 µL of TDE1 enzyme (Illumina 20034197) in TD buffer for a total of 50 µL. Samples were incubated for 10 min at 55°C and incubated with 50 mM EDTA for 30 min at 50°C, and then the supernatant was removed. Beads were washed twice with 50 mM EDTA and then Tween wash buffer for 2 min at 55°C. Beads were washed once with 10 mM Tris (pH 7.5) before PCR.

For all samples, the resulting beads were resuspended in 50 µL 1× NEBNext ultra II Q5 MasterMix with 500 nM Nextera oligos. Samples were incubated for 5 min at 72°C and 1 min at 98°C; followed by eight cycles of 15 sec at 98°C, 30 sec at 63°C, and 1 min at 72°C; with a final extension for 5 min at 72°C. Samples were then purified for sequencing on a NovaSeq 6000 in 150-bp paired-end mode.

### Data analysis

#### ATAC-seq

Reads were trimmed to remove adapter sequences using cutadapt (version 3.5) (Martin 2011). Trimmed reads were then mapped to hg38 using Bowtie 2 with the options “‐‐very-sensitive -k 20” (version 2.4.4) (Langmead and Salzberg 2012). Peaks were then called using Genrich (version 0.6.1) (https://github.com/jsh58/Genrich), a peak caller purpose-built to handle ATAC-seq data including accounting for duplicate and multimapping reads with the options “-j -y -r -e chrM -v.” Peaks were merged across all ATAC-seq samples. Overlapping peaks were merged to generate a consensus set of peaks using BEDTools merge (version 2.30.0) (Quinlan and Hall 2010). BAM files were filtered to remove reads mapping to mitochondria and to remove PCR duplicates using Picard markduplicates (version 2.25.6). Peaks were then quantified using filtered BAM files with BEDTools multicov. Differential accessible regions were identified using DESeq2 (version 4.1.2) (Love et al. 2014) to compare peaks in each infected cell line with those in uninfected Jurkat cells. Regions were considered differentially expressed if they had an adjusted *P-*value <0.05 and a log_2_ fold change greater than one. Filtered BAM files were used to generate CPM normalized bigWig plots using deepTools (version 3.5.1) (Ramírez et al. 2016). In the case of the CRISPR ATAC samples, visualization files were scaled according to the fraction of reads in peaks for that sample as previously described (Corces et al. 2018). Plotting was performed using ggplot2s (version 3.3.6) (Wickham 2009) and Integrative Genomics Viewer (IGV; version 2.11.2) (Robinson et al. 2011). Differentially accessible peaks were also output for each cell line and analyzed for motif differences using HOMER (version 4.11) (Heinz et al. 2010).

#### 4C-seq

Reads were trimmed in paired-end mode to remove primer sequences from the 5′ end of reads associated with the target window (read 1, NTTCTCTCTCAGGGTCATCCATTCCATG; read 2, GAGGGGCGGCGACTGGTGAGTACGCCAAAAATT) and filtered to select only reads with the correct primer sequence using the cutadapt option “‐‐discard-untrimmed.” Trimmed reads were then mapped to hg38 using Bowtie 2. The genome was segmented into restriction enzyme fragment windows corresponding to the first restriction enzyme (NlaIII) using the HiC-Pro utility script “digest_genome.py.” The resulting BED file was used to quantify fragment coverage across the genome and in each sample using BEDTools multicov. In parallel, BAM files were used to generate bigWig files using deepTools. Results were visualized using R (version 4.2) (R Core Team 2022) and IGV. Fragments of significant interaction defined by two replicates were defined using peakC with a window size of 21 fragments, within 1 Mb of the integration site (version GitHub as of November 2021) (Geeven et al. 2018). Genes were plotted using ggbio (version 1.36.0) (Yin et al. 2012).

Binarization and fragment quantification were performed as follows in R. The quantification file was loaded into R, and the quantification for each sample was binarized by setting the data point to “one” if at least 10 reads mapped to that fragment or to zero otherwise. A threshold of 10 reads was chosen to overcome observed index hopping in the NovaSeq platform based on the number of reads present in the uninfected Jurkat sample. Index hopping is a known sequencing artifact impacting patterned flow cell platforms (e.g., NovaSeq 6000 and HiSeq 4000). Index hopping (Kircher et al. 2012) occurs because of flow cell swapping of index sequences between libraries and impacts between 0.1% and 2% of reads. As this library should only have off-target amplification, any reads that passed on target filters represented index hopping from other libraries. We choose binarization with this cutoff because our experimental procedure required a large amount of amplification, and we did not have a UMI or sheer point to differentiate independent interaction events. We used a sliding window approach similar to those previously used (Melamed et al. 2018). We first set a set of overlapping windows of 40-fragment lengths (equivalent to 10,240 bp on average for a 4-bp recognition site restriction enzyme), which were overlapping each other by 35 fragments (each window was “slid” five fragments forward, corresponding to 1280 bp on average for a 4-bp recognition site restriction enzyme). We then summed the overlapping peaks for each 4C-seq sample to get the number of unique fragments detected (out of a maximum of 40 for the fragment window) in that set of windows. This smoothened representation was then used for plotting and for identifying overlapping regions of ATAC-seq sample.

#### Joint ATAC-seq 4C-seq data motif analysis

Overlapping regions of 4C-seq signal and ATAC-seq peaks were extracted using R, selecting only ATAC-seq peaks which were on (1) the same chromosome as the integration of interest and (2) overlapped with 4C-seq windows in which at least one replicate detected at least 10 of the possible 40 fragments. Peaks were filtered to remove regions overlapping encode blacklist regions (Amemiya et al. 2019), a set of commonly overrepresented genomic regions. Regions across the cell lines were analyzed separately and also as a pooled set of peaks. HOMER was used to detect motif enrichment within those regions relative to their expected frequency in the genome. Motifs were considered significantly enriched if the adjusted *P-*value was below 0.05. HOMER was used to output motif images and to annotate significantly interacting peaks with top enriched motifs for visualization in IGV.

#### DOGMA-seq

Reads from transcriptome and ATAC-seq libraries were aligned to a concatenated hg38 and NL43-d6-GFP HIV genome using Cell Ranger ARC (10x Genomics, version 2.0.1). The resulting matrices were analyzed using Seurat (version 4.1.1) (Hao et al. 2021) and Signac (version 1.7.0) (Stuart et al. 2021). Clones were demultiplexed using the Seurat function HTOdemux, after which doublets and negative cells were removed. Cells were filtered to have fewer than 100,000 ATAC fragments, fewer than 25,000 RNA UMIs, greater than 1000 ATAC fragments, greater than 1000 RNA UMIs, a nucleosome signal less than two, and a transcription start site enrichment greater than one. After quality control, SCTransform (version 0.3.5) (Hafemeister and Satija 2019) was used to normalize RNA-seq data. Separate 10x Genomics runs were integrated using FASTMNN on RNA data, a component of the batchelor package (version 1.10.0) (Haghverdi et al. 2018). The first 20 dimensions of the corrected mutual nearest-neighbor space were used to generate a mutual nearest-neighbor graph to find clusters (resolution of 0.5) and calculate a UMAP dimensionality reduction.

After clustering, peaks were recalled using MACS2 (version 2.2.7.1) (Zhang et al. 2008) through the Signac function “CallPeaks” for each cluster. Peaks identified by MACS2, the regions downstream from the HIV-1 provirus, and the proviral accessibility itself were then quantified using the Signac function “FeatureMatrix.” Pearson's correlations between HIV-1 accessibility, HIV gene expression, and accessibility downstream from HIV-1 integration were calculated using R. Plotting of correlations was completed with ggplot2. chromVAR (version 1.16.0) (Schep et al. 2017) was performed using Signac with motifs from the R package JASPAR 2020 (version 0.99.10) (Fornes et al. 2020) with default parameters. Significantly differentially active motifs associated with cell lines were identified using FindAllMarkers in Seurat using a Wilcoxon rank-sum test with the “rowMeans” replacing the default mean.fxn parameter.

#### Hi-C, H3K27ac HiChIP

Reads were mapped to a concatenated hg38 and NL43 genome, filtered, quantified into contact matrices, and quality controlled using HiC-Pro (version 3.1.0) (Servant et al. 2015) with the default parameters. Briefly, the HiC-Pro pipeline aligns each paired read to the genome separately and then filters the resulting mapped files to remove singletons, multimapping reads, low MAPQ containing reads, and unmapped reads. Filtered reads were then paired and analyzed to remove reads pairs that are not consistent with expected HiC read-pair configuration (e.g., two reads mapping in the same direction on one chromosome). Reads were then quantified for each restriction fragment in the genome to quantify interaction frequency. Contact matrices were converted to HIC files using the Hi-C Pro utility “hicpro2juicebox.sh.” KR (Knight and Ruiz 2013) normalized plotting was completed in R using plotgardener (version 1.2.10) (Kramer et al. 2022) at regions surrounding the HIV-1 integration site and regions without an HIV-1 integration. Data were further compared in a joint pairwise manner for Loess normalization and statistical comparison using HiCcompare (version 1.20.0) (Stansfield et al. 2018) with a resolution of 25 kb within a 1-Mb window of the integration site. Interacting regions were filtered, aggregated, and annotated in R.

## Data access

All raw and processed sequencing data generated in this study have been submitted to the NCBI Gene Expression Omnibus (GEO; https://www.ncbi.nlm.nih.gov/geo/) under accession number GSE230314.

## Supplementary Material

Supplement 1

Supplement 2

Supplement 3

Supplement 4
