# Supplementary material for: Integration site–dependent HIV-1 promoter activity shapes host chromatin conformation
Source: Genome Res. 2023 Jun;33(6):891–906. doi: 10.1101/gr.277698.123 (PMC10519397; doi:10.1101/gr.277698.123)
Supplement: Supplement 4 [file Supplemental_Figures.pdf]

SUPPLAMENTAL FIGURES

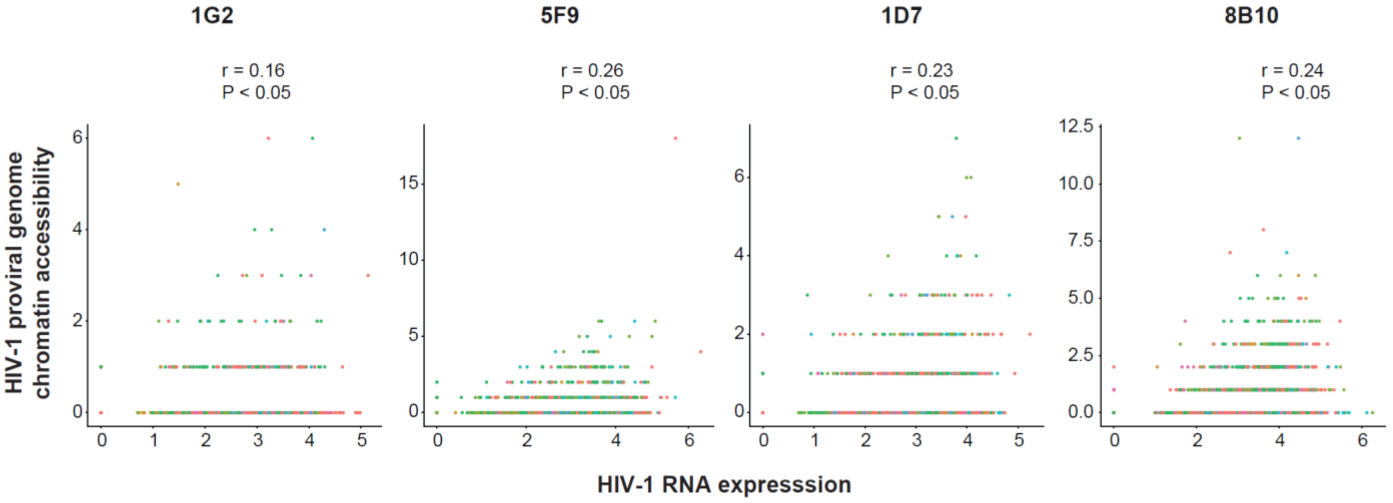

Figure S1. Correlations between HIV-1 RNA expression levels and HIV-1 genome chromatin accessibility. Correlation coefficient (r) and P values were determined by Pearson correlation in each cell line across all cells in that line.

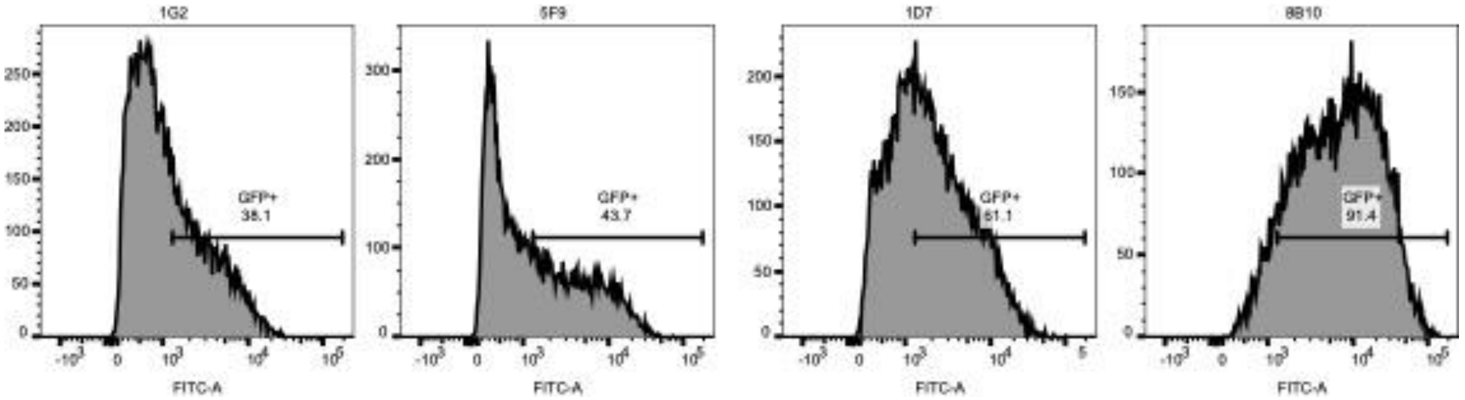

Figure S2. HIV-1 GFP expression is heterogenous across cell line clones. FACS plots showing the GFP+ of cell line clones, ordered from least to most chromatin accessibility.

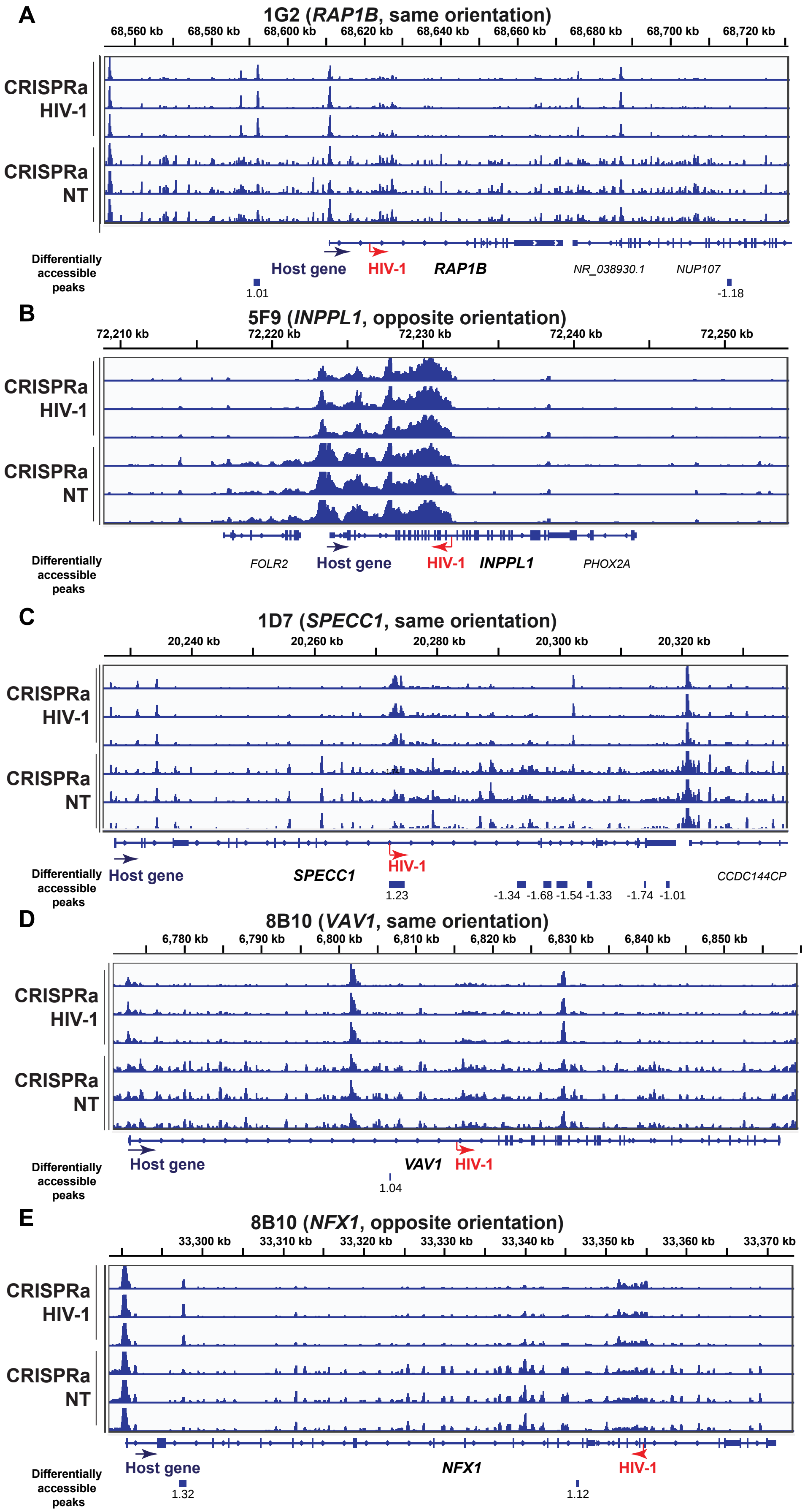
A
1G2 (RAP1B, same orientation)
68,560 kb 68,580 kb 68,600 kb 68,620 kb 68,640 kb 68,660 kb 68,680 kb 68,700 kb 68,720 kb
CRISPRa HIV-1
CRISPRa NT
Differentially accessible peaks
Host gene
HIV-1
RAP1B
NR_038930.1
NUP107
1.01
-1.18
B
5F9 (INPPL1, opposite orientation)
72,210 kb 72,220 kb 72,230 kb 72,240 kb 72,250 kb
CRISPRa HIV-1
CRISPRa NT
Differentially accessible peaks
FOLR2
Host gene
HIV-1
INPPL1
PHOX2A
C
1D7 (SPECC1, same orientation)
20,240 kb 20,260 kb 20,280 kb 20,300 kb 20,320 kb
CRISPRa HIV-1
CRISPRa NT
Differentially accessible peaks
Host gene
SPECC1
HIV-1
CCDC144CP
1.23
-1.34 -1.68 -1.54 -1.33 -1.74 -1.01
D
8B10 (VAV1, same orientation)
6,780 kb 6,790 kb 6,800 kb 6,810 kb 6,820 kb 6,830 kb 6,840 kb 6,850 kb
CRISPRa HIV-1
CRISPRa NT
Differentially accessible peaks
Host gene
VAV1
HIV-1
1.04
E
8B10 (NFX1, opposite orientation)
33,300 kb 33,310 kb 33,320 kb 33,330 kb 33,340 kb 33,350 kb 33,360 kb 33,370 kb
CRISPRa HIV-1
CRISPRa NT
Differentially accessible peaks
Host gene
NFX1
HIV-1
1.32
1.12

Figure S3. Impact of HIV-1-targetted CRISPR activation across cell lines. Genomic tracks and differentially accessible regions in CRISPRa expressing cell lines with an HIV-1-targetting guide RNA or a nontargeting guide RNA in (A) 1G2, (B) 5F9, (C) 1D7, or (D-E) 8B10. Significance determined by DESeq2 Tracks are CPM normalized and scaled to the fraction of reads in peaks, a range of 0-3 is shown on each track. Each peak is annotated with its Log2 fold change relative to the nontargeting control.

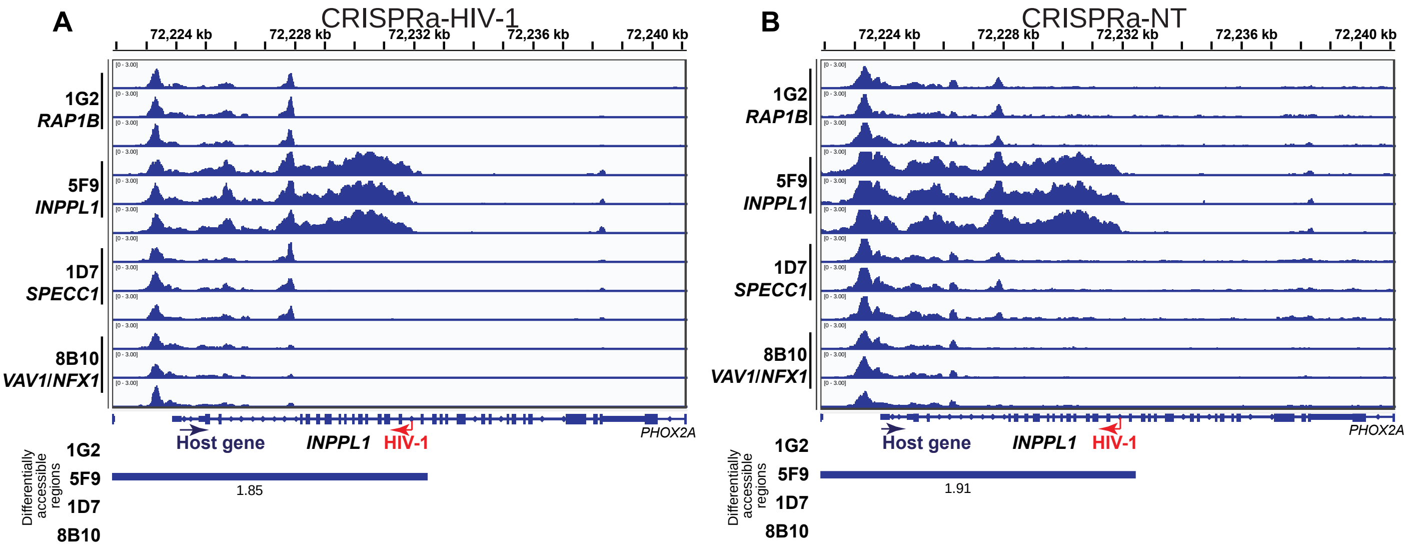

Figure S4. HIV-1-targetting CRISPRa fails to increase HIV-1-associated host chromatin accessibility. The chromatin accessibility and significantly differentially accessible regions across cell lines at the *INPPL1* gene in CRISPRa-transduced cell lines with an (A) HIV-1-targetting guide RNA or (B) a nontargeting guide RNA.

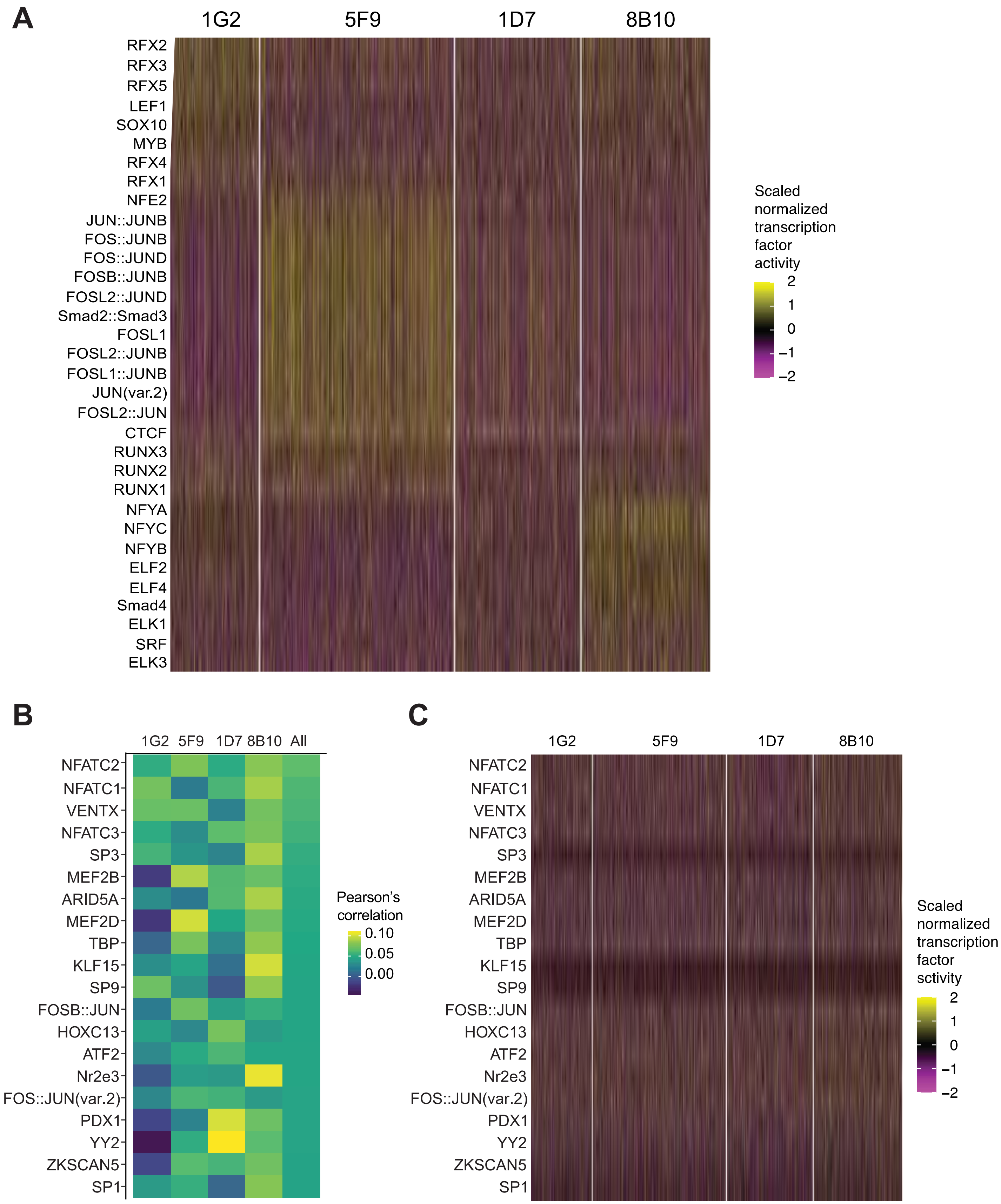
A
1G2
5F9
1D7
8B10
RFX2
RFX3
RFX5
LEF1
SOX10
MYB
RFX4
RFX1
NFE2
JUN::JUNB
FOS::JUNB
FOS::JUND
FOSB::JUNB
FOSL2::JUND
Smad2::Smad3
FOSL1
FOSL2::JUNB
FOSL1::JUNB
JUN(var.2)
FOSL2::JUN
CTCF
RUNX3
RUNX2
RUNX1
NFYA
NFYC
NFYB
ELF2
ELF4
Smad4
ELK1
SRF
ELK3
Scaled normalized transcription factor activity
2
1
0
−1
−2
B
1G2
5F9
1D7
8B10
All
NFATC2
NFATC1
VENTX
NFATC3
SP3
MEF2B
ARID5A
MEF2D
TBP
KLF15
SP9
FOSB::JUN
HOXC13
ATF2
Nr2e3
FOS::JUN(var.2)
PDX1
YY2
ZKSCAN5
SP1
Pearson's correlation
0.10
0.05
0.00
C
1G2
5F9
1D7
8B10
NFATC2
NFATC1
VENTX
NFATC3
SP3
MEF2B
ARID5A
MEF2D
TBP
KLF15
SP9
FOSB::JUN
HOXC13
ATF2
Nr2e3
FOS::JUN(var.2)
PDX1
YY2
ZKSCAN5
SP1
Scaled normalized transcription factor sctivity
2
1
0
−1
−2

Figure S5. Correlations between transcription factor activity and HIV-1 chromatin accessibility differ in cell line clones having different HIV-1 integration sites independent of clone-clone differences. HIV-1 chromatin accessibility was measured by DOGMA-Seq. Transcription factor activity was measured by ChromVar. (A) Transcription factor activity of the top 10 positively enriched transcription factors in each cell line. (B) Heatmap of the Pearson correlation between transcription factor activities and HIV-1 chromatin accessibility. Pearson correlation calculated using R. Top 20 motifs by average correlation across cell lines shown, ordered by decreasing aggregate average correlation. (C) Transcription activity of the same 20 high correlation motifs across cell lines.

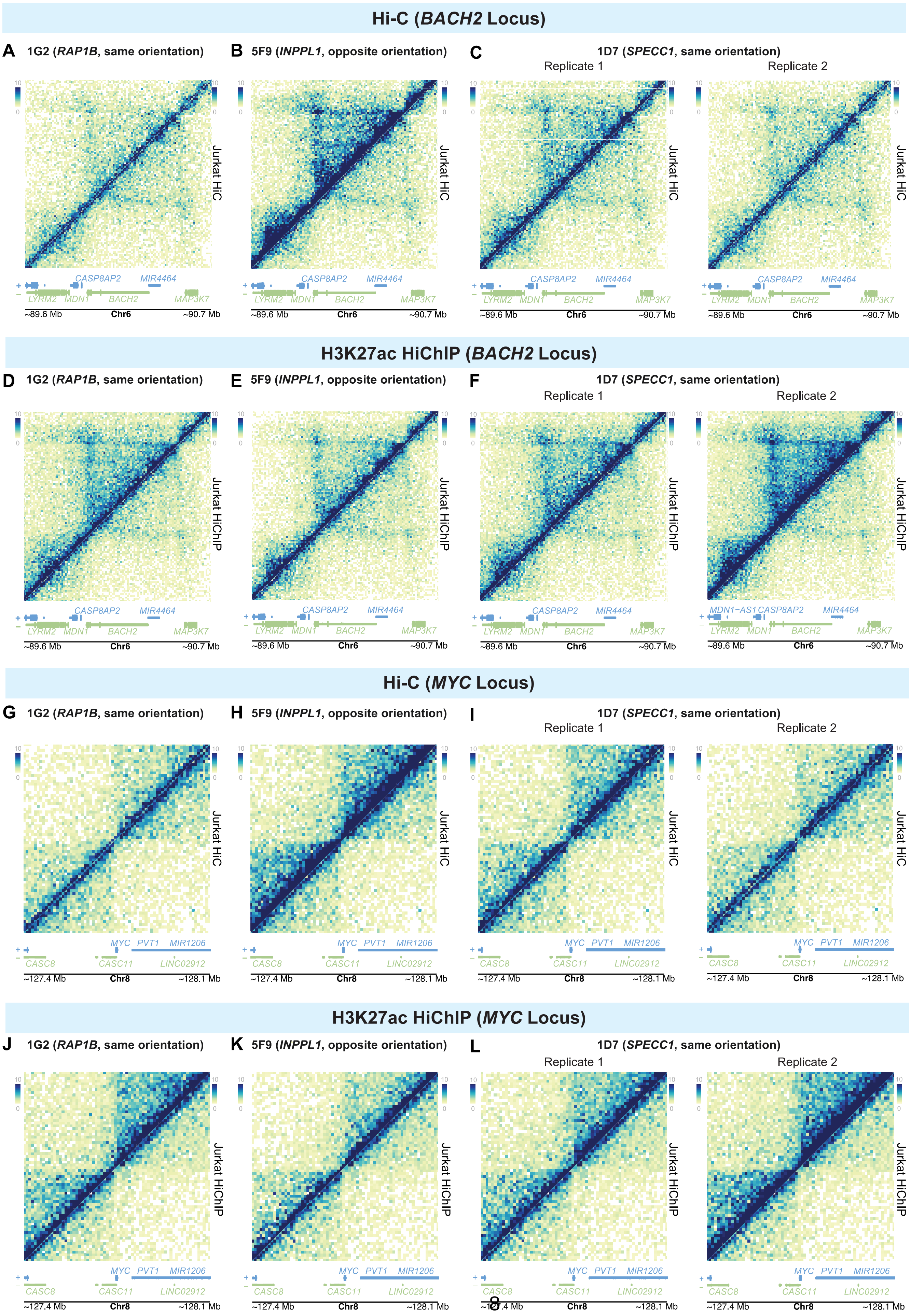
Hi-C (BACH2 Locus)
A 1G2 (RAP1B, same orientation)
B 5F9 (INPPL1, opposite orientation)
C 1D7 (SPECC1, same orientation)
Replicate 1
Replicate 2
Jurkat HiC
CASP8AP2
MIR4464
LYRM2
MDN1
BACH2
MAP3K7
~89.6 Mb
Chr6
~90.7 Mb
H3K27ac HiChIP (BACH2 Locus)
D 1G2 (RAP1B, same orientation)
E 5F9 (INPPL1, opposite orientation)
F 1D7 (SPECC1, same orientation)
Jurkat HiChIP
MDN1-AS1
Hi-C (MYC Locus)
G 1G2 (RAP1B, same orientation)
H 5F9 (INPPL1, opposite orientation)
I 1D7 (SPECC1, same orientation)
MYC
PVT1
MIR1206
CASC8
CASC11
LINC02912
~127.4 Mb
Chr8
~128.1 Mb
H3K27ac HiChIP (MYC Locus)
J 1G2 (RAP1B, same orientation)
K 5F9 (INPPL1, opposite orientation)
L 1D7 (SPECC1, same orientation)

Figure S6. Impact of HIV-1 on genome wide 3D chromatin conformation and H3K27ac enhancer loops at the heterologous sites. Heatmap shows Kight-Ruiz (KR) normalized signal at a 10 kb resolution surrounding BACH2 (A-F) and MYC (G-L) by Hi-C (A–C, G-I) and H3K27ac HiChIP (D–F, J-L).

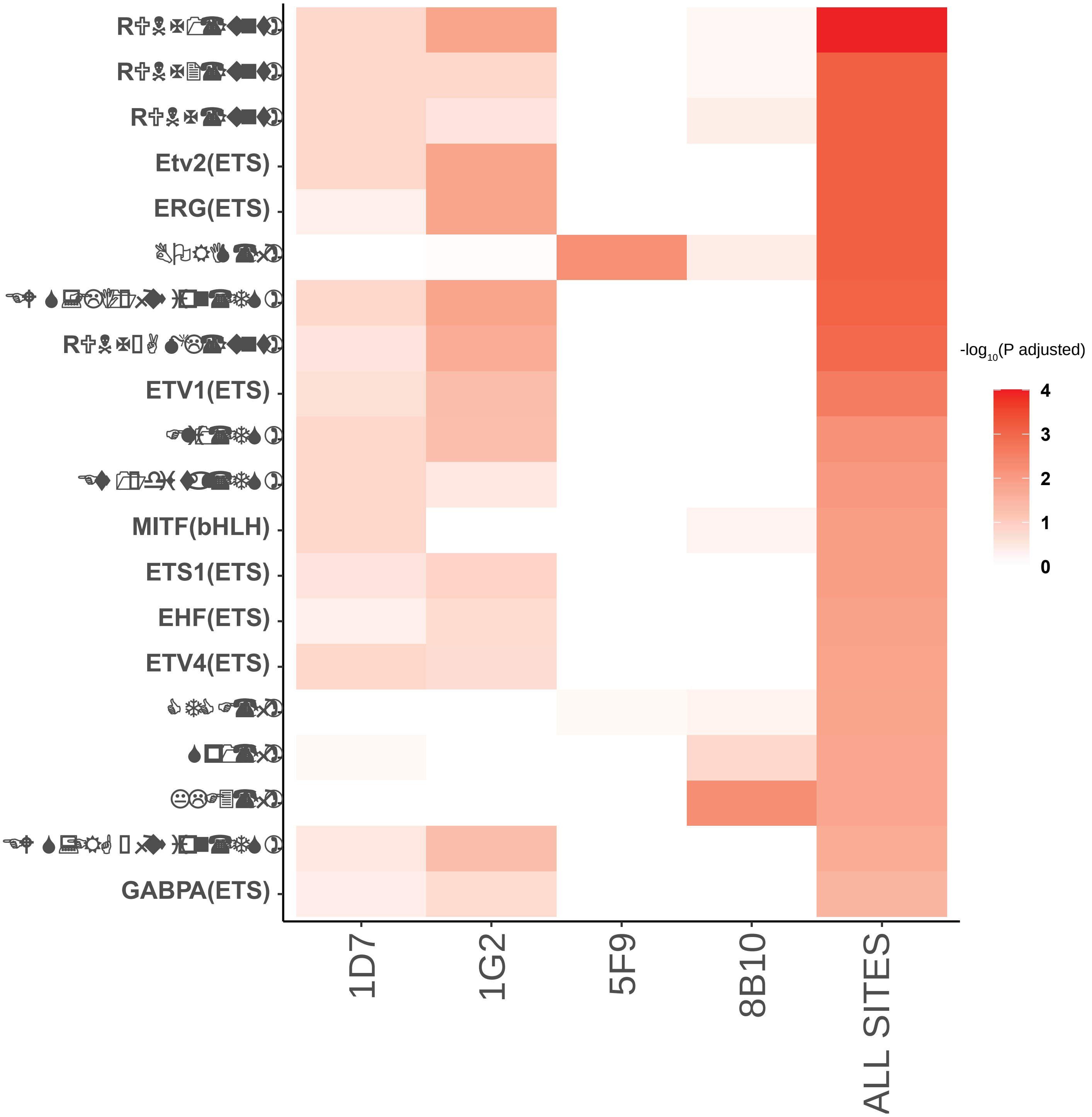

Figure S7. Transcription factor binding motifs enriched in both increased chromatin accessibility and HIV-1 host-chromatin interactions across cell lines. As in figure 6, HIV-1 interacting peaks, defined as regions of overlapping ATAC-seq peaks and 4C-seq signal were extracted from the integrated chromosome in each cell line. Transcription factor binding motifs were identified in each set of peaks separately, and across peaks by HOMER. Top known motifs ranked by adjusted P values.
